# Comparing the MicroRNA Spectrum between Serum and Plasma

**DOI:** 10.1371/journal.pone.0041561

**Published:** 2012-07-31

**Authors:** Kai Wang, Yue Yuan, Ji-Hoon Cho, Sara McClarty, David Baxter, David J. Galas

**Affiliations:** 1 Institute for Systems Biology, Seattle, Washington, United States of America; 2 Luxembourg Center for Systems Biomedicine, University of Luxembourg, Luxembourg City, Luxembourg; South Texas Veterans Health Care System and University Health Science Center San Antonio, United States of America

## Abstract

MicroRNAs (miRNAs) are small, non-coding RNAs that regulate various biological processes, primarily through interaction with messenger RNAs. The levels of specific, circulating miRNAs in blood have been shown to associate with various pathological conditions including cancers. These miRNAs have great potential as biomarkers for various pathophysiological conditions. In this study we focused on different sample types’ effects on the spectrum of circulating miRNA in blood. Using serum and corresponding plasma samples from the same individuals, we observed higher miRNA concentrations in serum samples compared to the corresponding plasma samples. The difference between serum and plasma miRNA concentration showed some associations with miRNA from platelets, which may indicate that the coagulation process may affect the spectrum of extracellular miRNA in blood. Several miRNAs also showed platform dependent variations in measurements. Our results suggest that there are a number of factors that might affect the measurement of circulating miRNA concentration. Caution must be taken when comparing miRNA data generated from different sample types or measurement platforms.

## Introduction

Measuring the levels of specific analytes in bodily fluids, especially serum or plasma prepared from blood, is the most commonly used method in diagnosis. It is relatively noninvasive and in certain cases, with proper training and supervision, can be self-administered by patients for disease management (e.g. a blood sugar test). However, most of the current blood biomarkers are inadequate in specificity and sensitivity for definitive disease diagnosis. One of the major foci in biomedical research in the past few decades has been to identify biomarkers, or panels of biomarkers, in body fluids with clear disease association. Most of these activities are centered on identifying protein-based biomarkers; however, promise is still unfulfilled.

MicroRNAs (miRNAs) are endogenous 17 to 23 nucleotide-long noncoding regulatory RNA molecules that modulate cellular messenger RNA (mRNA) and protein levels by interacting with specific mRNAs, usually at the 3′ untranslated region (UTR), through partial sequence complementation [1], [2]. Thus far, over 1,000 human miRNAs have been identified (miRBase, www.mirbase.org). Recently, a significant number of miRNAs have also been found outside of the cells, and the levels of some of these extracellular miRNAs in circulation have been linked to different pathophysiological conditions. Examples of this include the associations of miR-141 with prostate cancer, miR-499 with myocardial infarction, and miR-122 with drug-induced liver injury [3]–[7]. These findings raise the possibility of using the levels of specific miRNAs in circulation as biomarkers for different pathological conditions [8]–[12]. Compared to protein-based biomarkers, miRNA offers several advantages: the complexity of miRNA is much lower than that of proteins; the miRNAs are stable in various bodily fluids; the sequences of many miRNAs are conserved among different clinically relevant species; the expression of some miRNAs are restricted to specific tissues or biological stages. The levels of miRNAs can also be easily measured by various commonly used laboratory methods, including assorted signal amplification strategies [8].

Despite significant progress in identifying the association of specific circulating miRNAs in various diseases, factors that may affect the measurement of circulating miRNA concentrations have yet to be fully addressed. Previous reports on the exportation of miRNAs from cells raised the prospect that plasma and serum might exhibit some differences in their miRNA content. We obtained serum and corresponding plasma samples from 12 healthy individuals to investigate the possible effects of different types of blood sample preparation on miRNA measurement. Our results suggested that different blood sample preparation methods might affect the concentration measurement of individual circulating miRNAs.

## Results

Serum and plasma samples from 12 individuals, 6 males and 6 females, with ages ranging from 22 to 41, were used for our measurements. Various blood cell components were also collected from 6 (3 males and 3 females) of the 12 donors (Table 1). The general sample information and concentrations of isolated RNA are listed on Table 1. No significant association between RNA concentration with either the gender or the age of the donor was observed. However the sera showed a higher RNA concentration than the corresponding plasma samples (with a p value of 0.0312 calculated by two-sample t-test). WBC, RBC, and platelets had much higher RNA concentrations than either plasma or serum, as expected. Unlike the RNA from RBC or WBC, the pattern of platelet total RNA is similar to serum and plasma on bioanalyzer (Figure S1).

**Table 1:** General information on samples and measurement results.

Sample ID	Gender	Age	RNA Concentration (ng/mL)					Measurement Platform		Number of Detectable miRNA Species						
			Serum	Plasma	Platelets	Red Blood Cells	White Blood Cells	Taqman Panels	Exiqon Panels	Taqman qPCR Panels (Ct< = 35)		Exiqon qPCR Panels (Ct< = 35)				
										Serum	Plasma	Serum	Plasma	Platelets	Red Blood Cells	White Blood Cells
55-29512	female	35	97	25	142	3745	2418		X			185	200	341	454	374
55-29515	female	39	51	20	70	4371	1167		X			156	164	288	477	391
11-39685	female	41	32	17	75	3999	1249		X			194	201	285	450	397
11-39684	male	35	108	35	73	3491	1137		X			240	212	301	421	359
11-39686	male	41	37	21	57	2444	862		X			195	161	309	471	395
11-39691	male	41	80	32	42	2481	1618		X			123	123	280	457	374
ID-3	female	22	39	36				X	X	172	158	274	200			
ID-4	female	29	67	49				X		163	198					
ID-5	female	29	75	32				X	X	174	178	296	267			
ID-1	male	28	70	80				X	X	198	234	216	174			
ID-2	male	29	38	39				X	X	192	157	194	141			
ID-6	male	NA	97	40				X		139	137					

### Plasma and Serum Contained Significant Number of miRNAs

#### a) miRNA measured by Taqman card

**Figure 1:**
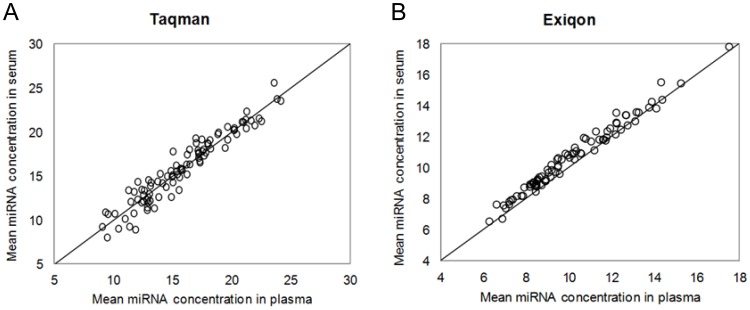
Comparing the individual miRNA concentrations between serum and plasma. The average concentrations (40-Ct values) of detected miRNAs in all serum and plasma samples within each platform were plotted – each point represents the average from all individuals of a specific miRNA. The Y-axis represents the average miRNA concentrations in serum and X-axis is the average concentration of corresponding plasma. The results from Taqman are showed in panel A and Exiqon are showed in panel B.

**Figure 2:**
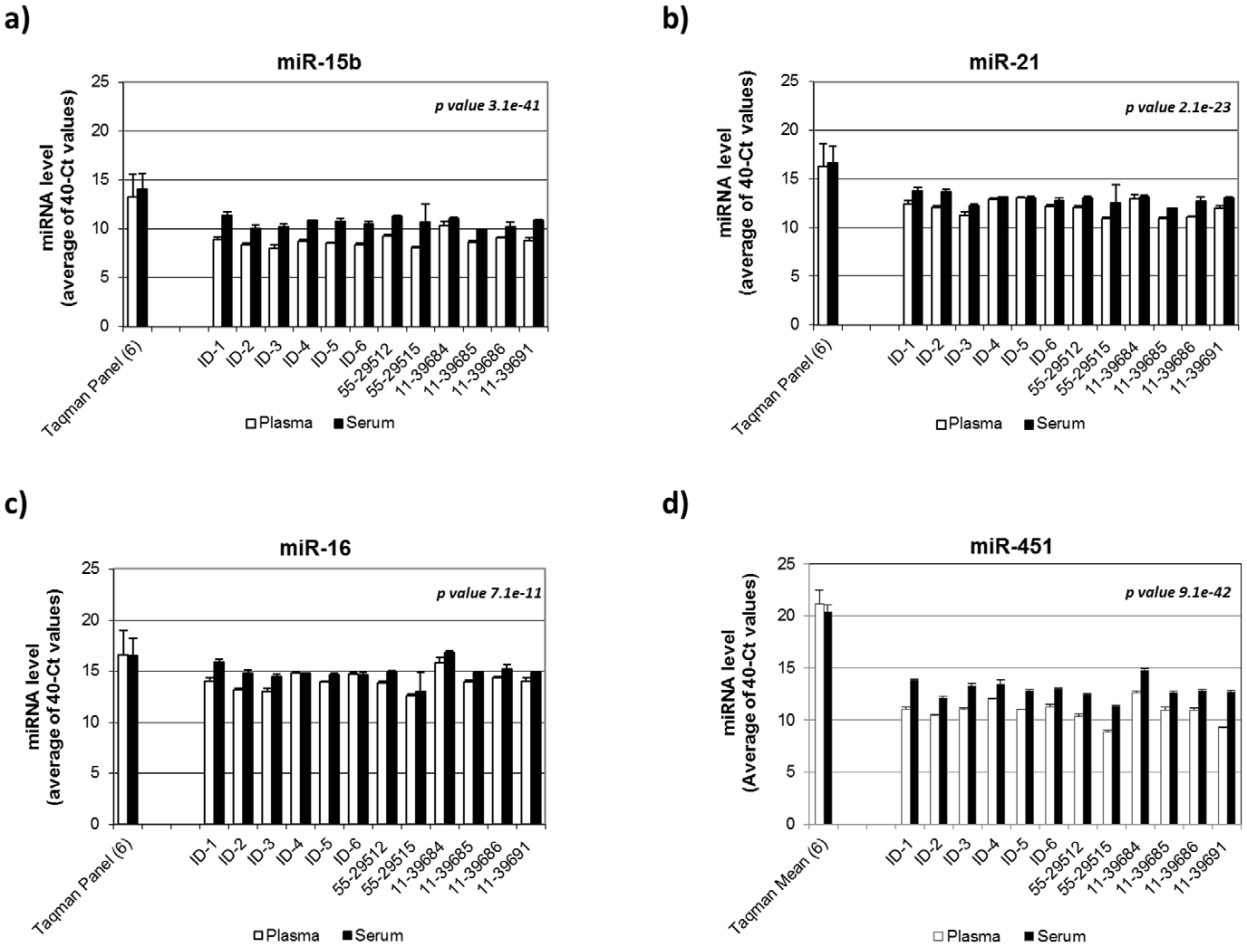
Examples of miRNA concentration differences between serum and plasma using individual Taqman QPCR primers. The sample IDs were listed on the X-axis and the miRNA concentrations were displayed on the Y-axis (in 40-Ct value). The miRNA IDs were indicated on top of the graph. Open bars represent plasma samples and the solid bars represent the corresponding serum samples. The values of standard derivation were obtained from three independent measurements. The original measurement results from Taqman card showed higher 40-Ct values since a pre-amplification step was employed. Two-way ANOVA was used to determine the statistical significance of the miRNA concentration differences between serum and plasma (p-values are shown in the figure).

The serum and plasma miRNA spectrum from 6 normal individuals (3 males and 3 females) was profiled with Taqman miRNA panel (Table 1). The number of detectable miRNA species from these samples ranged from 137 (in ID-6 plasma) to 234 (in ID-1 plasma) (Table 1). 106 miRNAs could be seen in all plasma samples, 118 in all serum samples, and 98 miRNAs could be seen in all plasma and serum samples tested (Table S1). No significant correlation between the number of detectable miRNA species with either the gender of the donor, the type of sample used (serum or plasma), or the RNA concentration was observed. Based on the 40-Ct values, there is no statistically significant difference between serum and plasma’s miRNA concentrations (p-value = 0.3991 from two-sample t-test) (Table S1 and Figure 1A). However, the serum samples always showed higher concentrations than plasma when we measured specific miRNA levels with individual QPCR Taqman primers regardless of the results from the initial Taqman card profiling, examples are shown in Figure 2. Based on the Taqman card measurement results, the most abundant miRNA species was miR-223 for all the serum samples and one of the plasma samples tested (Table S3).

The coefficient of variation (standard deviation/mean of concentration) for individual miRNA across samples from different donors was determined. Among the 6 plasma samples, miR-101 showed the highest concentration variation and miR-185 had the most consistent concentration (Table 2). In sera, miR-576-3p and miR-345 had the highest and lowest concentration variations, respectively. Interestingly, the levels of MammU6, RNU44 (SNORD 44) and RNU48 (SNORD48) which had been used to normalize miRNA concentration measurements were not consistently detected in the serum and plasma samples tested. In addition, the concentrations of these RNAs also showed significant variations among samples (Table S1).

**Table 2:** The list of miRNAs with the most and least concentration variations in serum and plasma samples and measurement platforms.

Platform	Taqman				Exiqon			
Sample	Plasma		Serum		Plasma		Serum	
Variability	miRNA ID#	Variability*	miRNA ID#	Variability*	miRNA ID#	Variability*	miRNA ID#	Variability*
**High**	miR-101	0.3095	miR-576-3p	0.3164	miR-22	0.2216	**miR-29c**	0.2424
	miR-376c	0.3002	**miR-532-5p**	0.2904	**miR-199a-3p**	0.2048	miR-106b	0.2400
	miR-155	0.2965	miR-18b	0.2723	miR-199a-5p	0.1939	miR-424	0.2115
	**miR-532-5p**	0.2732	miR-10a	0.2419	miR-328	0.1920	miR-148a	0.2093
	**miR-302c**	0.2445	**miR-302c**	0.2364	**miR-29c**	0.1897	**miR-199a-3p**	0.2074
**Low**	miR-18a	0.0492	miR-324-3p	0.0307	miR-346	0.0837	miR-1979	0.0806
	miR-320a	0.0468	miR-126	0.0297	miR-486-5p	0.0826	**miR-92a**	0.0780
	miR-106b	0.0400	miR-320a	0.0295	miR-16	0.0820	miR-1974	0.0712
	**miR-345**	0.0364	miR-19b	0.0282	**miR-720**	0.0738	miR-934	0.0584
	miR-185	0.0304	**miR-345**	0.0270	**miR-92a**	0.0644	**miR-720**	0.0563

* Coefficient of variation (standard deviation/mean) was used to measure the variability.

# Common miRNA species between serum and plasma were listed in boldface characters.

#### b) miRNA measured by Exiqon panels

**Figure 3:**
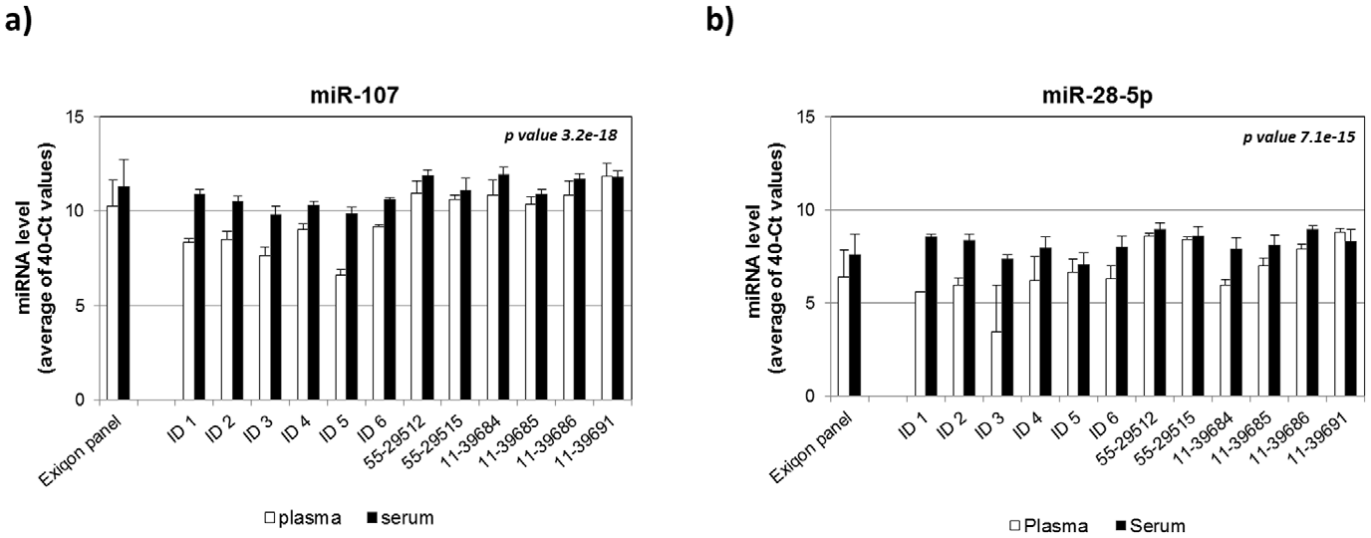
Examples of miRNA concentration differences between serum and plasma using individual Exiqon QPCR primers. The sample IDs were listed on the X-axis and the miRNA concentrations were displayed on the Y-axis (in 40-Ct value). The miRNA IDs were indicated on top of the graph. Open bars represent plasma samples and the solid bars represent the corresponding serum samples. The values of standard derivation were obtained from three independent measurements. Two-way ANOVA was used to determine the statistical significance of the miRNA concentration differences between serum and plasma (p-values are shown in the figure).

With Exiqon QPCR panels, the number of detectable miRNAs among different samples ranged from 123 to 296, with an average of 181 in plasma and 204 in serum. Among them, 90 miRNAs were shared in all plasma samples, 99 in all serum samples, and 83 in both (Table 1 and Table S2). Similar to Taqman results, no significant association between the numbers of detectable miRNA in relation to either gender or RNA concentrations was observed. Serum has more detectable miRNAs than plasma (Table 1). Most of the miRNAs in serum showed higher concentrations than the corresponding plasma samples (with a p-value = 0.0379 from two-sample t-test) (Table S2 and Figure 1B). The higher concentration of miRNAs in serum samples was also demonstrated with individual QPCR primers on some of the miRNAs, examples are shown in Figure 3. Except for one serum sample (sample 55-29515), miR-451 was the most abundant miRNA species based on the results of Exiqon miRNA QPCR panel (Table S3).

The coefficient of variation was used to determine the concentration variation of miRNAs measured by Exiqon panels. In plasma, miR-22 showed the highest concentration variation while miR-92a had the most stable concentration across samples. In serum, miR-29c and miR-720 had the highest and lowest concentration variations, respectively (Table 2). Like the Taqman miRNA measurement results, U6 and some SNORD RNAs were not consistently detected in both serum and plasma samples tested (Table S2).

### The Results between Two QPCR Platforms Showed Low Consistency

Samples from 4 individuals were profiled with both Taqman and Exiqon miRNA QPCR panels. Based on the primer annotations, 358 miRNAs were in common between Taqman card A and Exiqon plate I and II. After combining the two datasets, 67 miRNAs could be detected in all 4 serum and plasma samples by both platforms. The average miRNA concentration of the 67 commonly detectable miRNAs showed 6.7 Ct values higher in Taqman than the Exiqon measurements. This was probably caused by the pre-amplification step implemented in the Taqman miRNA measurement protocol. Within each platform, both Taqman and Exiqon exhibited high correlations between serum and corresponding plasma samples (based on the 40-Ct values) (Figure S2A). However, the 67 commonly detectable miRNAs in both plasma and serum samples showed very low correlations between the two platforms (Figure S2B).

**Figure 4:**
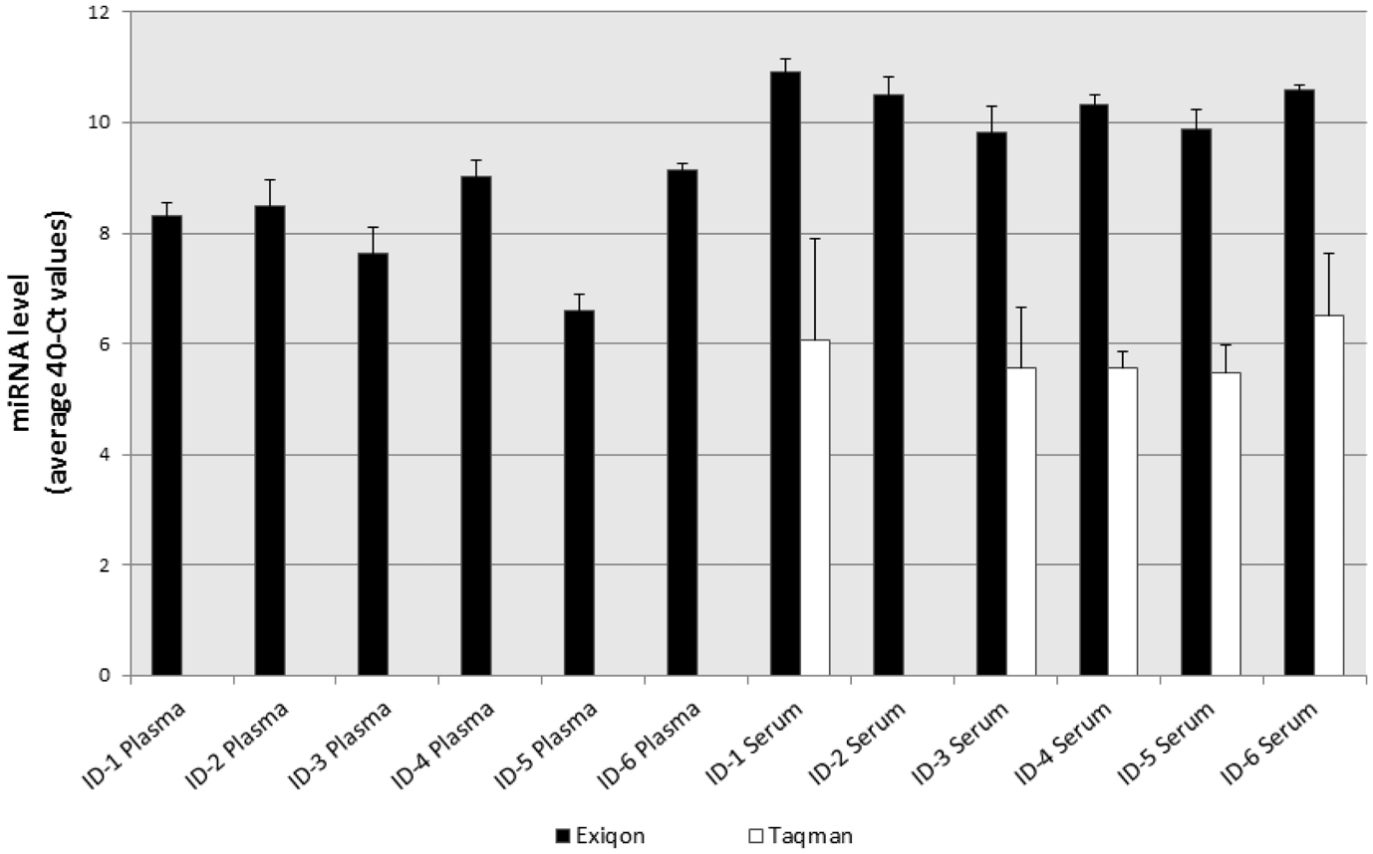
Certain miRNAs showing different measurement efficiency between measurement platforms. The sample IDs and type of samples were listed on the X-axis. The miRNA concentrations were displayed on the Y-axis (in 40-Ct value). Gray bars represent Exiqon measurement results while solid bars represent the Taqman measurements from the same sample. The values of standard derivation were obtained from three independent measurements.

When comparing the measurements between platforms, there were several miRNAs that showed platform-associated amplification; for example, miR-107 showed a much better amplification with Exiqon compared to Taqman (Figure 4 and Tables S1 and S2).

### Blood Cells Components Contained a Significant Amount of Detectable miRNAs

As expected, the blood cells had more detectable miRNA species, ranging from 280 to 477, compared to their serum or plasma counterparts (Tables 1 and Table S2). A significant number of these miRNAs (206 of them) were shared among all blood cell components. Either miR-223 or miR-451 was the most abundant miRNA in all RBC, WBC and platelet samples (Table S3). It is interesting to note that the concentration of miR-223 was relatively low in RBC compared to WBC and platelets. As in serum and plasma, U6 and SNORD RNAs were not detected in all samples, especially in platelets (Table S2). The degree of concentration variations of individual miRNAs in the WBC, RBC and platelet samples from different donors was also examined, and. the most variable and the most stable miRNA species were listed, respectively in Table 3.

**Table 3:** The list of miRNAs with the most and least concentration variations in different blood cell samples and measurement platforms.

Platform	Exiqon					
Sample	WBC		RBC		Platelets	
Variability	miRNA ID	Variability*	miRNA ID	Variability*	miRNA ID	Variability*
**High**	miR-33a	0.2246	miR-520b	0.2578	miR-379*	0.2494
	miR-136	0.2245	miR-33a	0.2515	miR-379	0.2300
	miR-101*	0.2053	miR-517c	0.2184	miR-369-5p	0.2232
	miR-543	0.1964	miR-338-3p	0.2169	miR-190	0.2227
	miR-96	0.1846	miR-100	0.2150	miR-654-3p	0.2218
**Low**	miR-30e	0.0172	miR-26a	0.0076	miR-423-5p	0.0368
	miR-27b	0.0161	miR-15b	0.0070	miR-130a	0.0363
	miR-23a	0.0152	miR-92a	0.0066	miR-210	0.0329
	miR-93*	0.0151	miR-19b	0.0066	miR-720	0.0284
	miR-548c-5p	0.0135	miR-30b	0.0063	miR-1974	0.0164

* Coefficient of variation (standard deviation/mean) was used to measure the variability.

# Common miRNA species between serum and plasma were listed in boldface characters.

### A Number of miRNAs are Presented in Specific Blood Components

Of the 742 miRNAs in Exiqon panels, 178 were never detected in any of the samples tested including serum, plasma and their corresponding blood cell components. There were 206 miRNA species that can be detected in all blood cell components (WBC, RBC and Platelets) and 80 were in all samples tested (WBC, RBC, Platelets, serum and plasma). Based on our criteria, there were 26 miRNAs can only be seen in RBC samples and 1 miRNA unique to WBC (miR-24-5p) (Tables S2 and S4).

## Discussion

There is a more than two-fold difference in the number of detectable miRNA species between the lowest (123 detectable miRNA in serum or plasma sample from 11-39691 with Exiqon panel) and highest (296 detectable miRNA in serum sample from ID-5 with Exiqon panel) number among serum and plasma samples tested (Table 1). However, no correlation between the number of detected miRNAs and the concentration of RNAs obtained was observed (Table 1). Therefore, the common practice of using similar amounts of RNAs might not be the best approach for circulating miRNA measurements. The difference in the number of detectable miRNA in the samples may have some physiological implications. However, it is difficult to interpret the significance of this difference without more detailed clinical information about the normal donors.

For miRNA measurement, data normalization is still a challenge, especially for circulating miRNA. Several small RNA species, such as U6 and SNORD RNAs have been used in miRNA data normalization. However, they are either not detectable or have very high concentration variations across different samples in our study (Tables S1 and S2). This observation suggests that RNAs like U6 or SNORD might not be suitable for normalizing extracellular miRNA measurements.

The levels of tissue-specific miRNAs in circulation have been advanced as potential blood biomarkers for various pathologies in specific organs. For example, the liver-specific miR-122 in drug-induced liver injury [12], [13], the muscle-specific miR-133a and b in muscle injury [13] and heart-specific miR-208 and miR-499 in myocardial infarction [5], [14]. Most of these miRNAs are not routinely detectable in the normal serum or plasma samples used in this study (Table S1). This supports the possibility of using the levels of these organ-specific miRNAs in circulation as biomarkers for site-specific pathologies.

### RNA Including miRNAs May be Released from Blood Cells into Serum During Coagulation Process

Since the serum and plasma were collected at the same time from the same individual, the higher RNA concentration in serum (in 10 out of 12 samples) (Table 1) suggests that additional RNA was released from cells during the coagulation process. Cell lysis, especially the highly abundant red blood cells, during the coagulation process is one of the plausible explanations for this RNA concentration difference between serum and plasma. However, the concentrations of some of the highly abundant miRNA species in blood cell components such as, miR-150 in WBCs, miR-16 in RBCs and miR-126 in platelets were almost the same between serum and plasma (Table S2). In addition, the miRNA concentration difference between serum and plasma showed some associations with miRNA spectrum in platelets and WBCs, instead of the most abundant cell population, RBCs, in the blood (Figure S3).

This RNA concentration difference between serum and plasma is consistent with the idea of RNA/miRNA ‘trafficking” between cellular compartments and the extracellular environment [7], [15]–[21]. During the coagulation process, blood cells are exposed to a “stressful environment” which may “stimulate” the release of certain miRNAs and other RNAs, as was observed when cells were exposed *in vitro* to serum-free conditions [7]. In addition, the platelets also contained significant amounts of RNA (Table 1) [22] which might also be released into the serum during the coagulation process.

The difference of molecular composition between serum and plasma is well documented [23]–[27]. For example, the coagulation process increases sample-to-sample variations on the observed proteome which makes data analysis and comparison difficult. Our miRNA study yields a similar conclusion and suggests that plasma may be the sample of choice in studying circulating miRNA, since RNA released during the coagulation process may change the true repertoire of circulating miRNA. When using archived samples in circulating miRNA studies, special attention must be given to this difference, since the majority of the archived samples are stored as serum.

### The Concentration for Some miRNAs may be Affected by Gender and the Measurement Platform

Gender-associated molecular differences have been reported in serum and platelet proteomes recently [28]–[30]. In addition to the concentration differences of some miRNAs between serum and plasma, a number of miRNAs showed concentration differences between genders. For example, miR-130b and miR-18b have slightly higher concentrations in male serum samples measured by Taqman platform (Table S1). This gender-associated circulating miRNA concentration difference has also been observed recently [31]. Even though the biological implications of these differences are yet to be determined, we cannot exclude the possibility that they are involved in some gender-associated functions.

The poor agreement among different miRNA measurement platforms has been reported earlier [32], [33]. The observation of low measurement consistency between the two commonly used QPCR miRNA measurement platforms and miRNA concentration differences between serum and plasma further illustrate the need for a commonly accepted miRNA measurement platform and sample preparation method, so that cross-study comparisons and independent data validations can be performed more effectively.

While there are numerous encouraging reports on the use of specific circulating miRNAs as biomarkers for various pathological conditions, it is important to understand factors that might affect the measurement of extracellular miRNA concentration and take these explicitly into account in diagnostic protocols. Our results suggest that circulating miRNA levels may be affected by various intrinsic and extrinsic factors including: the gender of the donor, the measurement platform used, and type of samples obtained. Even though some of these intrinsic factors may be associated with specific biological activities; however, with limited samples used in the study, it is not our intention to further interpret the results for its biological implications. Our findings simply illustrate the need for a larger and more comprehensive study with additional serum and plasma samples from better controlled donors in order to establish the boundaries for the use of circulating miRNA-based biomarkers. In addition, the standardization for sample preparation and miRNA measurement method is urgently needed to further develop circulating miRNA based biomarker.

## Materials and Methods

### Sample and RNA Isolation

Samples were obtained from either Biological Specialty Corporation (Biological Specialty Corporation, Colmar, PA) or Innovative Research (Innovative Research, Novi, MI). All samples were collected from healthy individuals with proper approvals by institutional review boards. The serum, plasma, and blood cell fractions were prepared according to standard protocols. In brief, serum was prepared by leaving the blood at room temperature for one hour before harvesting the supernatant. Plasma, RBC, WBC and platelets were prepared from 30 mLs of EDTA blood. The blood was initially centrifuged at 1000× g for 15 minutes to separate the plasma and blood cells. The supernatant was carefully removed and centrifuged again at 2500× g for 15 minutes to separate plasma and platelets. The platelet pellet was then resuspended in 7 ml of phosphate buffered saline (PBS, 137 mM NaCl, 2.7 mM KCl, 4.3 mM Na_2_HPO_4_, 1.4 mM KH_2_PO_4_, pH 7.5). The buffy coat from the initial spin was carefully collected and resuspended in RBC lysis buffer (10 mM KHCO_3_, 150 mM NH_4_Cl and 0.1 mM EDTA pH8.0) to remove RBC. The WBC containing solution was then spun at 1000× g for 10 minutes to collect WBC. The cell pellet was resuspended in 7 mL of PBS. The RBC fraction was taken directly from the original cell pellet. For serum or plasma, total RNA was extracted from 100 µl of the sample using the miRNeasy kit (Qiagen, Valencia, CA) as previously described [6], [12]. For RBC, WBC and platelets, 50 µl of the resuspended cell pellets were used in RNA extraction. The extracted RNA was assessed for quality and quantity with Agilent 2100 Bioanalyzer (Agilent, Santa Clara, CA) and NanoDrop 1000 spectrophotometer (Thermo Scientific, Wilmington, DE).

### MicroRNA Measurement

For the Taqman assay (Applied Biosystems, Carlsbad, CA), 12 µl of isolated RNA was dried and resuspended in 3 µl of water. The cDNA was then generated using Megaplex RT Primer pools. Pre-amplification was performed using 2.5 µl cDNA according to the manufacturer’s protocol. The resulting samples were diluted and loaded onto the TaqMan® Array Human miRNA Panel. For Exiqon (Exiqon, Woburn, MA) miRNA profiling, 20 µl of RNA was used for cDNA synthesis using MiRCURY LNA™ Universal RT kit. The cDNA was then diluted and dispensed into the Exiqon Human miRNA panels. Since it is difficult to obtain accurate RNA concentrations we used the same volume of RNA isolated from serum and plasma samples in miRNA measurements. We only used card A in Taqman-based miRNA measurements, since it contained the majority of abundant miRNA species. The blood cell fractions were profiled with Exiqon miRNA panels only. QPCR was conducted with a 7900HT fast real-time PCR system (Applied Biosystems, Foster City, CA). Amplification results were analyzed with SDS 2.3 (Applied Biosystems, Foster City, CA). The amplification curves were individually inspected and miRNAs with abnormal amplification patterns or Ct values greater than 35 were removed from analysis. The complete Taqman and Exiqon QPCR miRNA profiling results are provided in Tables S1 and S2.

## Supporting Information

Figure S1 **The Bioanalyzer electropherogram converted gel like image of RNA isolated from different sample types.** The sample types are indicated on the top and the position of 18S and 28S RNAs are labeled by arrows.
(TIF)

Figure S2 **Comparing the similarity of miRNA spectrum between serum and plasma.** Scatter plots were used to demonstrate the similarity of miRNA concentrations between serum and plasma within the same measurement platform (A). The platform was indicated on top of the figure. Different platforms gave low correlation on concentration measurement (B) within either serum or plasma samples. The sample type was labeled on top of the figure. The plots were based on the average concentrations of 67 commonly detectable miRNA species in both Taqman and Exiqon QPCR platforms
(TIF)

Figure S3 **The miRNA concentration between serum and plasma showed some association with miRNA concentrations in platelets and WBC.** Scatter plots were used to demonstrate the correlation of miRNA concentration difference between serum and plasma and difference between platelets and plasma (A), WBC and plasma (B), and RBC and plasma (C). The average miRNA concentration differences between serum and plasma were represented on the Y-axis while the average differences between blood cell components and plasma were on X-axis. The plots were based on the average concentrations of 67commonly detectable miRNAs in all the samples.
(TIF)

Table S1 **The serum and plasma miRNA measured by Taqman cards.**
(XLS)

Table S2 **The serum and plasma miRNA measured by Exiqon miRNA panels.**
(XLS)

Table S3 **The list of top 5 most abundatn miRNAs in different samples.**
(XLS)

Table S4 **The list of miRNAs showing preferential expression pattern among different blood components.**
(XLS)

## References

[1] Almeida MI, Reis RM, Calin GA (2011) MicroRNA history: Discovery, recent applications, and next frontiers. Mutation research 717: 1–8. 21458467 10.1016/j.mrfmmm.2011.03.009

[2] Zhao S, Liu MF (2009) Mechanisms of microRNA-mediated gene regulation. Science in China Series C, Life sciences/Chinese Academy of Sciences 52: 1111–1116. 10.1007/s11427-009-0152-y 20016967

[3] Wang GK, Zhu JQ, Zhang JT, Li Q, Li Y, et al (2010) Circulating microRNA: a novel potential biomarker for early diagnosis of acute myocardial infarction in humans. European heart journal 31: 659–666. 20159880 10.1093/eurheartj/ehq013

[4] Mitchell PS, Parkin RK, Kroh EM, Fritz BR, Wyman SK, et al (2008) Circulating microRNAs as stable blood-based markers for cancer detection. Proceedings of the National Academy of Sciences of the United States of America 105: 10513–10518. 18663219 10.1073/pnas.0804549105 PMC2492472

[5] Adachi T, Nakanishi M, Otsuka Y, Nishimura K, Hirokawa G, et al (2010) Plasma microRNA 499 as a biomarker of acute myocardial infarction. Clinical chemistry 56: 1183–1185. 20395621 10.1373/clinchem.2010.144121

[6] Weber JA, Baxter DH, Zhang S, Huang DY, Huang KH, et al (2010) The microRNA spectrum in 12 body fluids. Clinical chemistry 56: 1733–1741. 20847327 10.1373/clinchem.2010.147405 PMC4846276

[7] Wang K, Zhang S, Weber J, Baxter D, Galas DJ (2010) Export of microRNAs and microRNA-protective protein by mammalian cells. Nucleic acids research 38: 7248–7259. 20615901 10.1093/nar/gkq601 PMC2978372

[8] Etheridge A, Lee I, Hood L, Galas D, Wang K (2011) Extracellular microRNA: A new source of biomarkers. Mutation research 717: 85–90. 21402084 10.1016/j.mrfmmm.2011.03.004 PMC3199035

[9] Schwarzenbach H, Hoon DS, Pantel K (2011) Cell-free nucleic acids as biomarkers in cancer patients. Nature reviews Cancer 11: 426–437. 21562580 10.1038/nrc3066

[10] Li LM, Hu ZB, Zhou ZX, Chen X, Liu FY, et al (2010) Serum microRNA profiles serve as novel biomarkers for HBV infection and diagnosis of HBV-positive hepatocarcinoma. Cancer research 70: 9798–9807. 21098710 10.1158/0008-5472.CAN-10-1001

[11] Kosaka N, Iguchi H, Ochiya T (2010) Circulating microRNA in body fluid: a new potential biomarker for cancer diagnosis and prognosis. Cancer science 101: 2087–2092. 20624164 10.1111/j.1349-7006.2010.01650.x PMC11159200

[12] Wang K, Zhang S, Marzolf B, Troisch P, Brightman A, et al (2009) Circulating microRNAs, potential biomarkers for drug-induced liver injury. Proceedings of the National Academy of Sciences of the United States of America 106: 4402–4407. 19246379 10.1073/pnas.0813371106 PMC2657429

[13] Laterza OF, Lim L, Garrett-Engele PW, Vlasakova K, Muniappa N, et al (2009) Plasma MicroRNAs as sensitive and specific biomarkers of tissue injury. Clinical chemistry 55: 1977–1983. 19745058 10.1373/clinchem.2009.131797

[14] Corsten MF, Dennert R, Jochems S, Kuznetsova T, Devaux Y, et al (2010) Circulating MicroRNA-208b and MicroRNA-499 reflect myocardial damage in cardiovascular disease. Circulation Cardiovascular genetics 3: 499–506. 20921333 10.1161/CIRCGENETICS.110.957415

[15] Vickers KC, Palmisano BT, Shoucri BM, Shamburek RD, Remaley AT (2011) MicroRNAs are transported in plasma and delivered to recipient cells by high-density lipoproteins. Nature cell biology 13: 423–433. 21423178 10.1038/ncb2210 PMC3074610

[16] Mittelbrunn M, Gutierrez-Vazquez C, Villarroya-Beltri C, Gonzalez S, Sanchez-Cabo F, et al (2011) Unidirectional transfer of microRNA-loaded exosomes from T cells to antigen-presenting cells. Nature communications 2: 282. 10.1038/ncomms1285 PMC3104548 21505438

[17] Biton M, Levin A, Slyper M, Alkalay I, Horwitz E, et al (2011) Epithelial microRNAs regulate gut mucosal immunity via epithelium-T cell crosstalk. Nature immunology 12: 239–246. 21278735 10.1038/ni.1994

[18] Akao Y, Iio A, Itoh T, Noguchi S, Itoh Y, et al (2011) Microvesicle-mediated RNA molecule delivery system using monocytes/macrophages. Molecular therapy : the journal of the American Society of Gene Therapy 19: 395–399. 21102562 10.1038/mt.2010.254 PMC3034851

[19] Iguchi H, Kosaka N, Ochiya T (2010) Secretory microRNAs as a versatile communication tool. Communicative & integrative biology 3: 478–481. 21057646 10.4161/cib.3.5.12693 PMC2974086

[20] Zomer A, Vendrig T, Hopmans ES, van Eijndhoven M, Middeldorp JM, et al (2010) Exosomes: Fit to deliver small RNA. Communicative & integrative biology 3: 447–450. 21057637 10.4161/cib.3.5.12339 PMC2974077

[21] Miura K, Miura S, Yamasaki K, Higashijima A, Kinoshita A, et al (2010) Identification of pregnancy-associated microRNAs in maternal plasma. Clinical chemistry 56: 1767–1771. 20729298 10.1373/clinchem.2010.147660

[22] Osman A, Falker K (2011) Characterization of human platelet microRNA by quantitative PCR coupled with an annotation network for predicted target genes. Platelets 22: 433–441. 21438667 10.3109/09537104.2011.560305

[23] Tammen H, Schulte I, Hess R, Menzel C, Kellmann M, et al (2005) Prerequisites for peptidomic analysis of blood samples: I. Evaluation of blood specimen qualities and determination of technical performance characteristics. Combinatorial chemistry & high throughput screening 8: 725–733. 16464159 10.2174/138620705774962508

[24] Rai AJ, Gelfand CA, Haywood BC, Warunek DJ, Yi J, et al (2005) HUPO Plasma Proteome Project specimen collection and handling: towards the standardization of parameters for plasma proteome samples. Proteomics 5: 3262–3277. 16052621 10.1002/pmic.200401245

[25] Tammen H, Schulte I, Hess R, Menzel C, Kellmann M, et al (2005) Peptidomic analysis of human blood specimens: comparison between plasma specimens and serum by differential peptide display. Proteomics 5: 3414–3422. 16038021 10.1002/pmic.200401219

[26] Omenn GS, States DJ, Adamski M, Blackwell TW, Menon R, et al (2005) Overview of the HUPO Plasma Proteome Project: results from the pilot phase with 35 collaborating laboratories and multiple analytical groups, generating a core dataset of 3020 proteins and a publicly-available database. Proteomics 5: 3226–3245. 16104056 10.1002/pmic.200500358

[27] Hsieh SY, Chen RK, Pan YH, Lee HL (2006) Systematical evaluation of the effects of sample collection procedures on low-molecular-weight serum/plasma proteome profiling. Proteomics 6: 3189–3198. 16586434 10.1002/pmic.200500535

[28] Eidelman O, Jozwik C, Huang W, Srivastava M, Rothwell SW, et al (2010) Gender dependence for a subset of the low-abundance signaling proteome in human platelets. Human genomics and proteomics : HGP 2010: 164906. 20981232 10.4061/2010/164906 PMC2958630

[29] Nedelkov D, Phillips DA, Tubbs KA, Nelson RW (2007) Investigation of human protein variants and their frequency in the general population. Molecular & cellular proteomics : MCP 6: 1183–1187. 17468123 10.1074/mcp.M700023-MCP200

[30] Miike K, Aoki M, Yamashita R, Takegawa Y, Saya H, et al (2010) Proteome profiling reveals gender differences in the composition of human serum. Proteomics 10: 2678–2691. 20480504 10.1002/pmic.200900496

[31] Duttagupta R, Jiang R, Gollub J, Getts RC, Jones KW (2011) Impact of cellular miRNAs on circulating miRNA biomarker signatures. PloS one 6: e20769. 21698099 10.1371/journal.pone.0020769 PMC3117799

[32] Sato F, Tsuchiya S, Terasawa K, Tsujimoto G (2009) Intra-platform repeatability and inter-platform comparability of microRNA microarray technology. PloS one 4: e5540. 19436744 10.1371/journal.pone.0005540 PMC2677665

[33] Ach RA, Wang H, Curry B (2008) Measuring microRNAs: comparisons of microarray and quantitative PCR measurements, and of different total RNA prep methods. BMC biotechnology 8: 69. 18783629 10.1186/1472-6750-8-69 PMC2547107

