# Supplementary figures and images for: Comparing the MicroRNA Spectrum between Serum and Plasma
Source: PLoS One. 2012 Jul 31;7(7):e41561. doi: 10.1371/journal.pone.0041561 (PMC3409228; doi:10.1371/journal.pone.0041561)

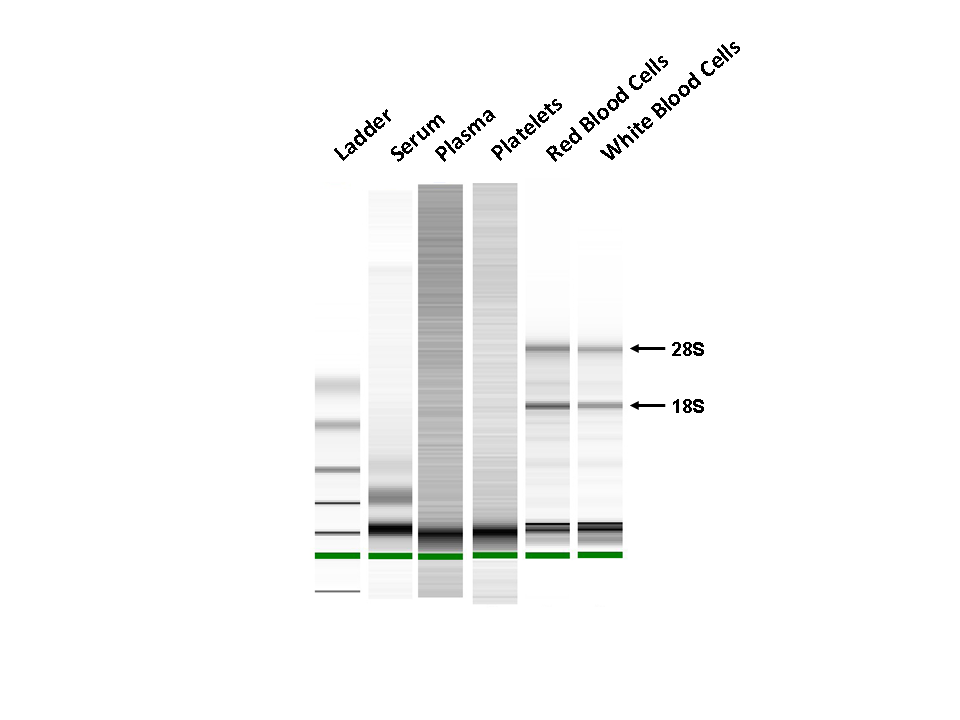

Supplement: Figure S1 — The Bioanalyzer electropherogram converted gel like image of RNA isolated from different sample types. The sample types are indicated on the top and the position of 18S and 28S RNAs are labeled by arrows. (TIF) [file pone.0041561.s001.tif]

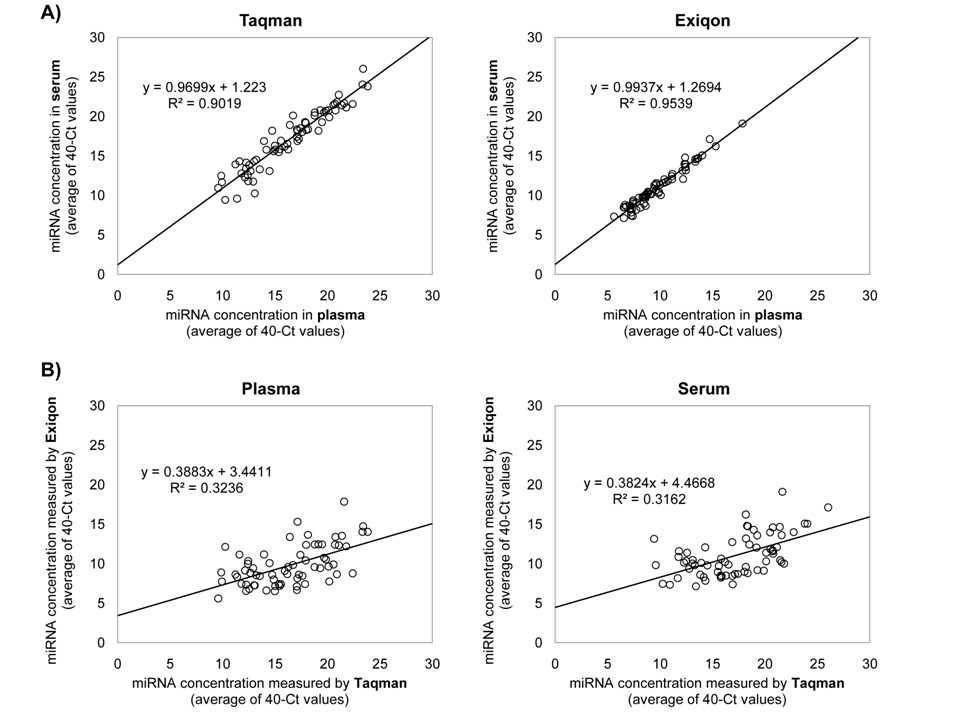

Supplement: Figure S2 — Comparing the similarity of miRNA spectrum between serum and plasma. Scatter plots were used to demonstrate the similarity of miRNA concentrations between serum and plasma within the same measurement platform (A). The platform was indicated on top of the figure. Different platforms gave low correlation on concentration measurement (B) within either serum or plasma samples. The sample type was labeled on top of the figure. The plots were based on the average concentrations of 67 commonly detectable miRNA species in both Taqman and Exiqon QPCR platforms (TIF) [file pone.0041561.s002.tif]

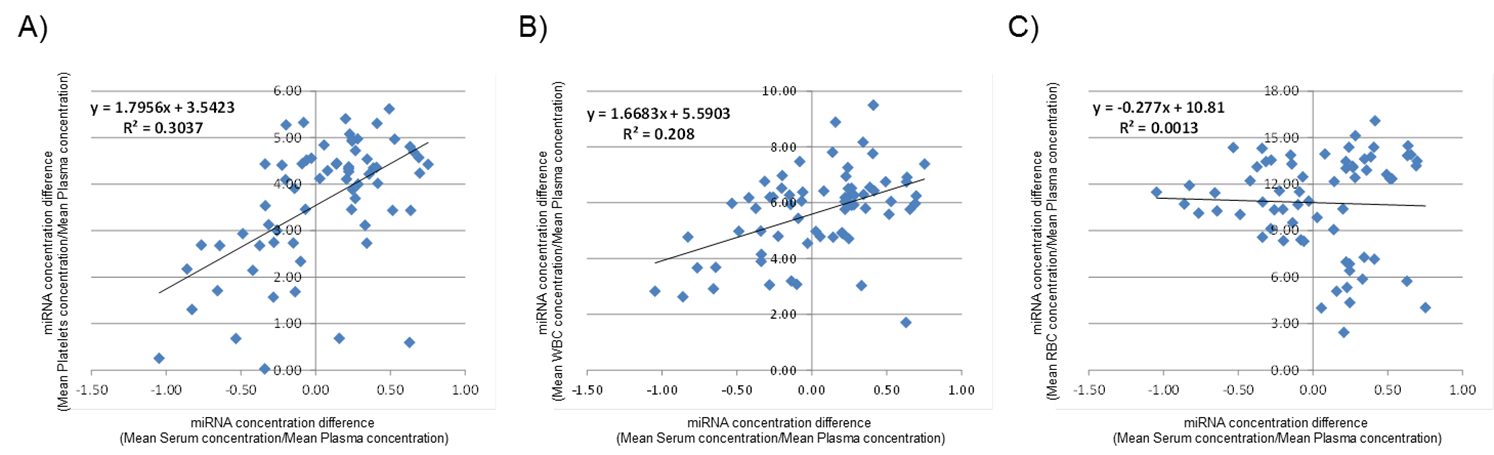

Supplement: Figure S3 — The miRNA concentration between serum and plasma showed some association with miRNA concentrations in platelets and WBC. Scatter plots were used to demonstrate the correlation of miRNA concentration difference between serum and plasma and difference between platelets and plasma (A), WBC and plasma (B), and RBC and plasma (C). The average miRNA concentration differences between serum and plasma were represented on the Y-axis while the average differences between blood cell components and plasma were on X-axis. The plots were based on the average concentrations of 67commonly detectable miRNAs in all the samples. (TIF) [file pone.0041561.s003.tif]
